# Vitamin D and resveratrol in sarcopenic obesity: a systematic review highlighting the gap in phenotype-defined randomized controlled trials

**DOI:** 10.3389/fnut.2026.1818450

**Published:** 2026-05-14

**Authors:** Cristina Russo, Maria Stella Valle, Sofia Surdo, Michele Tuttobene, Maria Teresa Cambria, Lucia Malaguarnera

**Affiliations:** 1 Department of Biomedical and Biotechnological Sciences, Section of Pathology, School of Medicine, University of Catania, Catania, Italy; 2 Laboratory of Neuro-Biomechanics, Section of Physiology, Department of Biomedical and Biotechnological Sciences, University of Catania, Catania, Italy; 3 Italian Center for the Study of Osteopathy (CSDOI), Catania, Italy; 4 ARNAS Garibaldi Hospital, Catania, Italy; 5 Department of Biomedical and Biotechnological Sciences, Section of Biochemistry, School of Medicine, University of Catania, Catania, Italy

**Keywords:** inflammation, mitochondrial dysfunction, precision nutrition, randomized controlled trials, resveratrol, sarcopenic obesity, vitamin D

## Abstract

**Background:**

Sarcopenic obesity (SO) is an inflammatory-metabolic condition characterized by the coexistence of excess adiposity and impaired skeletal muscle mass and function. Vitamin D and resveratrol modulate regulatory pathways implicated in SO pathophysiology, including NF-κB signaling, PGC-1α, mitochondrial regulation, and redox balance. Whether this mechanistic rationale has translated into phenotype-defined randomized clinical trials remains unclear.

**Methods:**

We conducted a systematic dual-track search across PubMed, Scopus, and Web of Science to identify randomized controlled trials (RCTs) evaluating isolated vitamin D or resveratrol supplementation in adults with explicitly defined sarcopenic obesity or meeting implicit SO phenotype criteria, defined as the concurrent presence of adiposity and objective muscle impairment at baseline. Trials lacking confirmation of both components at enrollment were excluded. Risk of bias was assessed using Cochrane RoB 2.0.

**Results:**

The search retrieved five records (PubMed *n* = 5; Scopus *n* = 0; Web of Science *n* = 0). Following full-text assessment, none met eligibility criteria requiring baseline confirmation of both adiposity and sarcopenia together with isolated vitamin D or resveratrol supplementation (*n* = 0). Retrieved RCTs in related populations did not simultaneously require adiposity and muscle impairment as enrollment criteria. As a result, no phenotype-defined interventional evidence specific to sarcopenic obesity was identified.

**Conclusion:**

Despite compelling mechanistic convergence, randomized interventional evidence in strictly defined sarcopenic obesity populations is currently lacking. Future RCTs must adopt phenotype-defined enrollment strategies integrating adiposity, muscle dysfunction, and mechanistic endpoints to determine whether micronutrient signaling can meaningfully modify outcomes in SO.

**Systematic review registration:**

https://www.crd.york.ac.uk/PROSPERO/view/CRD420261307248, identifier PROSPERO (CRD420261307248).

## 1 Introduction

Sarcopenic obesity (SO) is increasingly recognized as a distinct and clinically consequential phenotype defined by the coexistence of excess adiposity and impaired skeletal muscle mass and function (1). SO is characterized by the coexistence of excess adiposity and impaired skeletal muscle mass and function. Sarcopenia required confirmation of low muscle mass and/or impaired muscle strength or reduced physical performance according to established consensus-based diagnostic criteria, including the European Working Group on sarcopenia in Older People (EWGSOP2), the Asian Working Group for sarcopenia (AWGS), or the Foundation for the National Institutes of Health (FNIH) criteria (1–4).

Unlike classical sarcopenia, which primarily reflects age-related anabolic resistance and neuromuscular decline, SO develops within a metabolically dysregulated biological milieu characterized by insulin resistance, chronic low-grade inflammation, oxidative stress, and mitochondrial dysfunction (5).

Adipose tissue expansion promotes the release of pro-inflammatory cytokines and lipotoxic intermediates that activate Nuclear Factor Kappa B (NF-κB)-dependent transcriptional programs and Forkhead box O family transcription factors (FOXO)-mediated catabolic pathways, thereby accelerating proteolysis. Concurrent impairment of Peroxisome Proliferator-Activated Receptor Gamma Coactivator 1-Alpha (PGC-1α)-dependent mitochondrial biogenesis compromises oxidative capacity and muscle quality (6). Together, these alterations indicate that SO represents an integrated inflammatory-metabolic disorder rather than a simple coexistence of obesity and sarcopenia (7).

Within this inflammatory-mitochondrial network, micronutrient signaling has emerged as a biologically plausible modulatory axis (7). Vitamin D, through ligand-dependent activation of the vitamin D receptor (VDR), regulates transcriptional programs extending beyond calcium homeostasis and myogenic differentiation (8). VDR signaling can suppress NF-κB activity, influence oxidative stress responses, and interact with mitochondrial regulatory pathways potentially affecting PGC-1α–dependent biogenesis and metabolic homeostasis (9, 10). In parallel, resveratrol engages substantially overlapping regulatory pathways through a distinct upstream architecture. Activation of the Nicotinamide Adenine Dinucleotide (NAD^+^) Sirtuin 1 dependent deacetylase sirtuin-1 (SIRT1) and AMP-activated protein kinase (AMPK) promotes deacetylation and functional activation of PGC-1α, enhances mitochondrial biogenesis and oxidative phosphorylation, and attenuates inflammatory gene transcription via modulation of NF-κB. SIRT1–FOXO interactions further influence antioxidant defenses and proteostatic balance (11).

Thus, vitamin D and resveratrol have been reported to modulate several regulatory pathways that govern inflammation, mitochondrial integrity, and muscle protein turnover, including NF-κB, PGC-1α, FOXO, and networks associated with AMPK and SIRT1 activity. The key distinction lies not in their ultimate molecular targets, but in their regulatory entry points: vitamin D acts primarily through ligand-activated nuclear receptor–mediated transcriptional control, whereas resveratrol operates via NAD^+^-dependent deacetylase activation and energy-sensing cascades (12).

This partially overlapping regulatory architecture suggests that both compounds may influence related inflammatory and mitochondrial pathways relevant to SO, albeit through different upstream mechanisms.

These mechanistic nuances raise an important clinical consideration. Micronutrient interventions in SO may not be interchangeable; rather, their effectiveness could depend on the dominant biological driver within a given phenotype. Vitamin D may exert greater benefit in deficiency-defined muscle vulnerability, whereas resveratrol may be more aligned with metabolically inflamed SO characterized by heightened inflammatory burden and mitochondrial dysfunction (13). Such a precision phenotype-targeting framework implies that responsiveness may depend on baseline inflammatory status, metabolic dysregulation, and micronutrient deficiency rather than chronological age alone.

Despite the partial overlap in the mechanistic pathways influenced by these compounds, translation into phenotype-defined clinical research remains uncertain. Randomized trials have evaluated vitamin D in sarcopenia or obesity separately and resveratrol in metabolic or inflammatory conditions, yet the combined SO phenotype has rarely been operationally defined at enrollment. Failure to distinguish between sarcopenia, obesity, and their coexistence may obscure phenotype-specific efficacy and perpetuate translational ambiguity.

Accordingly, the present study was designed to determine whether randomized controlled trials (RCTs) have evaluated isolated vitamin D or resveratrol supplementation in populations rigorously defined as SO. We initially sought to perform quantitative synthesis; however, in the absence of eligible phenotype-defined trials, this review instead maps the available evidence and identifies structural gaps in trial design. By clarifying whether mechanistic rationale has translated into targeted clinical investigation, we aim to delineate priorities for future phenotype-guided randomized studies in SO.

## 2 Methods

### 2.1 Study design and reporting

This systematic review was conducted and reported in accordance with the Preferred Reporting Items for Systematic Reviews and Meta-Analyses 2020 (PRISMA 2020) guidelines (14) and the study protocol was prospectively registered in the International Prospective Register of Systematic Reviews (PROSPERO; registration number CRD420261307248).

The review was designed to determine whether RCTs have evaluated isolated vitamin D or resveratrol supplementation in adults with rigorously defined SO.

Given the anticipated scarcity of phenotype-defined trials, the protocol prespecified quantitative synthesis contingent upon the identification of eligible studies. In the absence of such trials, structured narrative synthesis and evidence mapping were planned.

### 2.2 Eligibility criteria

The review question was structured according to the Population-Intervention-Comparator-Outcome (PICO) framework. Studies were considered eligible if they met predefined methodological and phenotypic criteria. Only randomized controlled trials RCTs, including parallel-group or crossover designs, were considered. The population of interest comprised adults with SO confirmed at baseline. SO was defined as the concurrent presence of excess adiposity and objective sarcopenia. Adiposity was considered present when documented by body mass index (BMI), waist circumference, total or regional body fat mass, or measures of visceral adiposity. Sarcopenia required confirmation of low muscle mass and/or impaired muscle strength or reduced physical performance according to established consensus-based diagnostic criteria, including the European Working Group on Sarcopenia in Older People (EWGSOP2), the Asian Working Group for Sarcopenia (AWGS), or the Foundation for the National Institutes of Health (FNIH) criteria. When available, definitions of SO were evaluated with reference to the ESPEN/EASO consensus framework (1–4). Studies were considered eligible when the diagnostic criteria applied by the investigators were consistent with established consensus frameworks for sarcopenia or SO, even when minor methodological variations in body composition assessment or functional testing were present.

Eligible interventions consisted of isolated supplementation with vitamin D (including cholecalciferol, ergocalciferol, calcifediol, calcitriol, or active analogues) or isolated resveratrol formulations. Trials in which these compounds were administered as part of multi-component interventions were excluded unless the independent effect of the compound could be clearly isolated.

Studies conducted in obese populations without baseline confirmation of sarcopenia, or in sarcopenic populations without documented adiposity, were excluded from the primary phenotype-defined sarcopenic obesity evidence base.

### 2.3 Search strategy

A structured electronic search was performed across PubMed/MEDLINE, Scopus, and Web of Science (Core Collection) from database inception to the final search date (31 January 2026) (15).

Because SO is not consistently indexed, a phenotype-sensitive retrieval strategy was applied (Supplementary Table S1).

Search terms combined explicit SO terminology with constructs capturing the simultaneous presence of sarcopenia and obesity within the same record. Separate intervention-specific searches were conducted for vitamin D and for resveratrol. To ensure that retrieved studies explicitly evaluated the interventions of interest, search terms were restricted to title and abstract fields.

Filters identifying randomized controlled trials were applied using publication type indexing and free-text indicators of randomization or placebo control. Review articles and study protocols were excluded. No date restrictions were imposed. Database-specific syntax adaptations were applied across platforms (Supplementary Table S1). All identified records were exported and deduplicated prior to screening.

Because the objective of the review was to identify randomized controlled trials conducted in populations explicitly defined as SO, the search strategy was intentionally designed to prioritize phenotype specificity rather than broader retrieval of studies conducted in obesity or sarcopenia alone. This restrictive approach was adopted to enable phenotype-aligned evidence mapping and to avoid inclusion of trials in which SO was not formally characterized at baseline.

### 2.4 Study selection and data extraction

Titles and abstracts were independently screened by two reviewers to assess eligibility according to the predefined inclusion criteria. Potentially relevant studies proceeded to full-text evaluation. Full-text articles were assessed independently to determine whether they satisfied requirements for baseline confirmation of both adiposity and sarcopenia and for isolated vitamin D or resveratrol supplementation. Discrepancies were resolved through discussion and consensus.

For studies undergoing full-text assessment, data were extracted using a standardized template documenting study design, participant characteristics, baseline phenotype definition, adiposity and muscle-related criteria, intervention characteristics, comparator details, and reported outcomes. Information on mechanistic endpoints was recorded when available to contextualize biological relevance.

### 2.5 Risk of bias and certainty assessment

Risk of bias assessment using the Cochrane Risk of Bias 2.0 tool was prespecified for all eligible SO defined RCTs. Certainty of evidence evaluation using the Grading of Recommendations Assessment, Development and Evaluation (GRADE) framework was also planned.

However, because no studies met the eligibility criteria for phenotype-defined SO, formal risk-of-bias assessment and certainty grading were not performed.

### 2.6 Data synthesis

Quantitative meta-analysis was prespecified and would have been performed if eligible phenotype-defined RCTs had been identified. As no such trials were available, statistical pooling was not undertaken. Findings were synthesized narratively using an evidence-mapping approach. In addition to summarizing study characteristics, this framework was used to identify gaps in phenotype alignment across the retrieved literature, specifically evaluating whether randomized trials explicitly defined SO at baseline or instead investigated obesity and sarcopenia as separate conditions, and to assess the degree of alignment between the mechanistic rationale for vitamin D and resveratrol and the inflammatory-mitochondrial features characteristic of SO.

## 3 Results

### 3.1 Study selection and phenotype alignment

**Figure 1:**
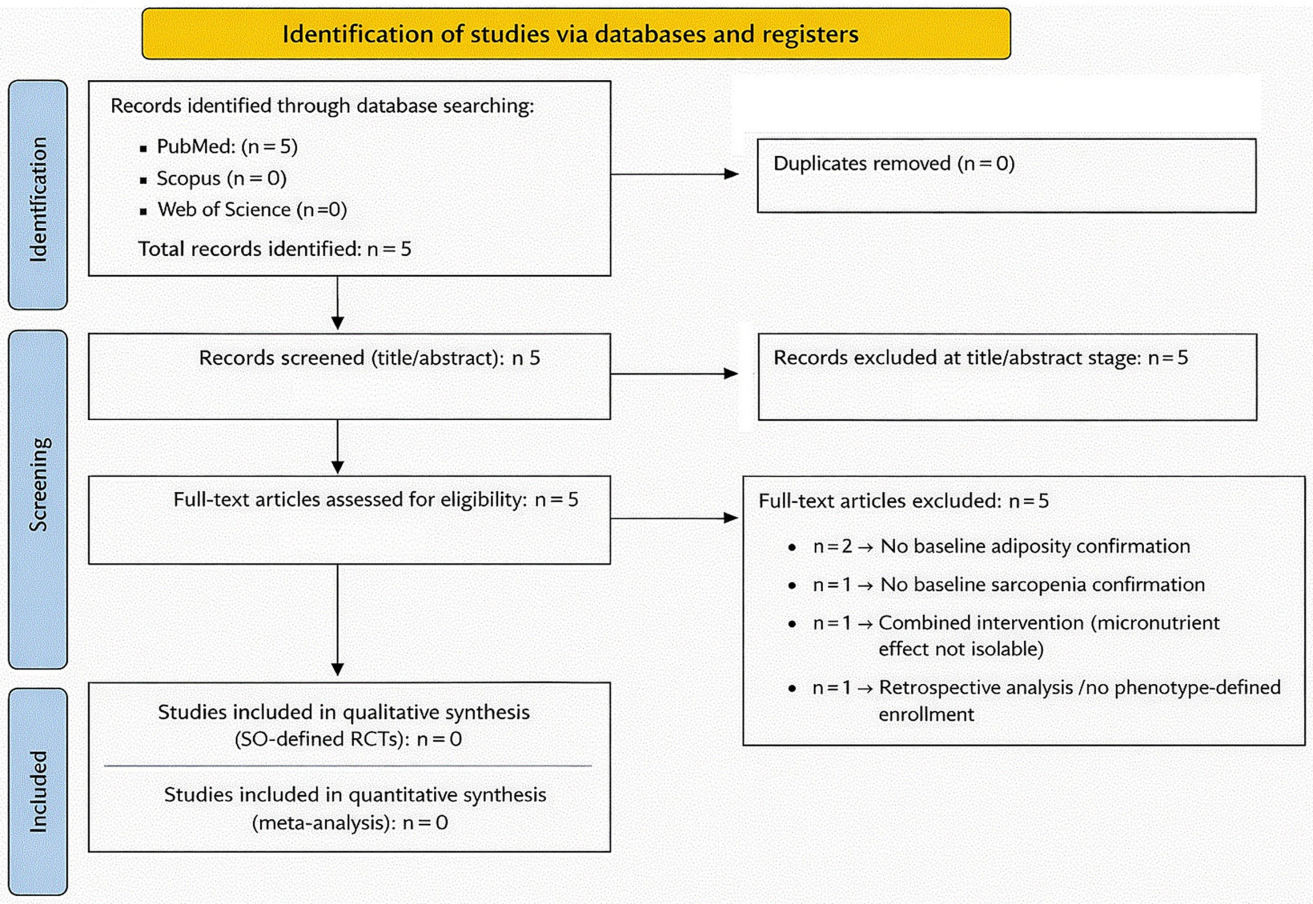
PRISMA 2020 flow diagram of study selection. Five records were identified through database searching (PubMed *n* = 5; Scopus *n* = 0; Web of Science *n* = 0). All full-text articles were excluded due to absence of phenotype-defined sarcopenic obesity enrollment or non-isolated intervention design. No randomized controlled trials were included in the review.

The phenotype-sensitive dual-track search strategy conducted across PubMed, Scopus, and Web of Science retrieved five records (16–20) all identified through PubMed. No additional records were identified in Scopus or Web of Science, and no duplicates were detected. The study selection process is detailed in the PRISMA 2020 flow diagram (Figure 1), which illustrates the identification, screening, eligibility, and exclusion steps. All five records progressed to full-text assessment following title and abstract screening. Upon detailed evaluation, none of the retrieved studies fulfilled the predefined eligibility criteria requiring baseline confirmation of both excess adiposity and objective sarcopenia within a rigorously defined SO phenotype, together with isolated supplementation of vitamin D or resveratrol. The five full-text randomized trials (16–20) reflected varying degrees of partial phenotype overlap. One study represented a retrospective analysis of a randomized trial conducted in individuals with obesity and metabolic disease; although sarcopenic obesity was referenced conceptually, participant selection was not based on combined adiposity and sarcopenia criteria. Two trials evaluated vitamin D supplementation in sarcopenic or pre-sarcopenic populations but did not require documented adiposity at baseline. Another trial enrolled participants described as having SO; however, the intervention combined structured exercise with nutritional supplementation, precluding isolation of the independent effect of vitamin D. A further study assessed high-dose vitamin D supplementation in older adults with Dual-Energy X-Ray Absorptiometry (DXA)-derived measures of body composition, yet SO was not prespecified as an inclusion criterion (Figure 1).

The characteristics of these five trials and the specific reasons for exclusion are summarized in Table 1. None simultaneously satisfied all predefined requirements of phenotype-defined enrollment and isolated micronutrient intervention. To further contextualize these findings, a structured phenotype-trial alignment analysis was performed (Table 2). This framework illustrates the fragmentation of the current evidence base. While individual components of SO, muscle mass, or muscle function were variably assessed across studies, no trial operationalized the full combined phenotype at enrollment while isolating vitamin D or resveratrol supplementation. The evidence landscape therefore reflects phenotype-adjacent investigation rather than phenotype-defined experimentation.

**Table 1:** Randomized controlled trials assessed at full text and reasons for exclusion.

Study (First author, year)	Population	Intervention	Baseline SO defined	Reason for exclusion
Nilsson et al. (16)	Adults with obesity and metabolic disease	Protein supplementation + resistance exercise (retrospective analysis of RCT)	No	Retrospective analysis; participants not enrolled based on combined adiposity and sarcopenia criteria
Jabbour et al. (17)	Older adults with DXA-derived indices of sarcopenia and obesity	High-dose vitamin D supplementation	No	Participants not recruited according to predefined SO diagnostic criteria
El Hajj et al. (18)	Pre-sarcopenic elderly adults	Vitamin D supplementation	No	No baseline confirmation of adiposity
Malafarina et al. (19)	Hip fracture patients with sarcopenia	Multi-nutritional supplementation	No	No adiposity requirement; multi-component intervention
Kim et al. (20)	Community-dwelling women with SO	Exercise + nutritional supplementation	Partially	Combined intervention; independent effect of vitamin D not isolable

Summary of the five RCTs identified through database searching and evaluated at full-text level. None fulfilled all predefined eligibility criteria requiring simultaneous baseline confirmation of excess adiposity and objective sarcopenia together with isolated vitamin D or resveratrol supplementation. Reasons for exclusion are reported to illustrate the phenotype misalignment and/or intervention design limitations observed across retrieved studies. SO, sarcopenic obesity; DXA, dual-energy X-ray absorptiometry.

**Table 2:** Phenotype–trial architecture alignment across retrieved randomized controlled trials.

Study (first author, year)	Adiposity confirmed at baseline	Sarcopenia confirmed at baseline	Isolated micronutrient intervention	Meets SO-defined enrollment	Structural limitation
Nilsson et al. (16)	Yes	No	No	No	Obesity-focused RCT; sarcopenia not operationalized; retrospective analysis
Jabbour et al. (17)	Yes	Not required for enrollment	Yes	No	Body composition assessed, but SO not used as inclusion criterion
El Hajj et al. (18)	No	Yes	Yes	No	Sarcopenia-focused RCT without adiposity requirement
Malafarina et al. (19)	No	Yes	No	No	Multi-nutrient intervention; no adiposity stratification
Kim et al. (20)	Yes	Yes	No	No	Combined exercise + supplementation; micronutrient effect not isolable

Structured evaluation of the degree to which retrieved RCTs operationalized baseline confirmation of adiposity and sarcopenia and implemented isolated micronutrient supplementation. The table illustrates the fragmentation of the current evidence base, showing that although individual components of SO were variably addressed, no trial fulfilled all criteria for phenotype-defined enrollment combined with isolated vitamin D or resveratrol intervention. SO, sarcopenic obesity.

No RCTs evaluating isolated resveratrol supplementation in rigorously defined SO were identified.

### 3.2 Risk of bias

Because no RCTs met eligibility criteria for phenotype-defined SO, formal risk-of-bias assessment using the Cochrane Risk of Bias 2.0 tool was not applicable to the primary evidence base. Consequently, structured domain-level appraisal and certainty grading were not performed.

### 3.3 Quantitative synthesis

Quantitative meta-analysis was prespecified contingent upon identification of eligible phenotype-defined randomized trials. As no such studies were identified, statistical pooling was not undertaken. The analysis therefore proceeded through structured narrative synthesis and evidence mapping.

## 4 Discussion

**Figure 2:**
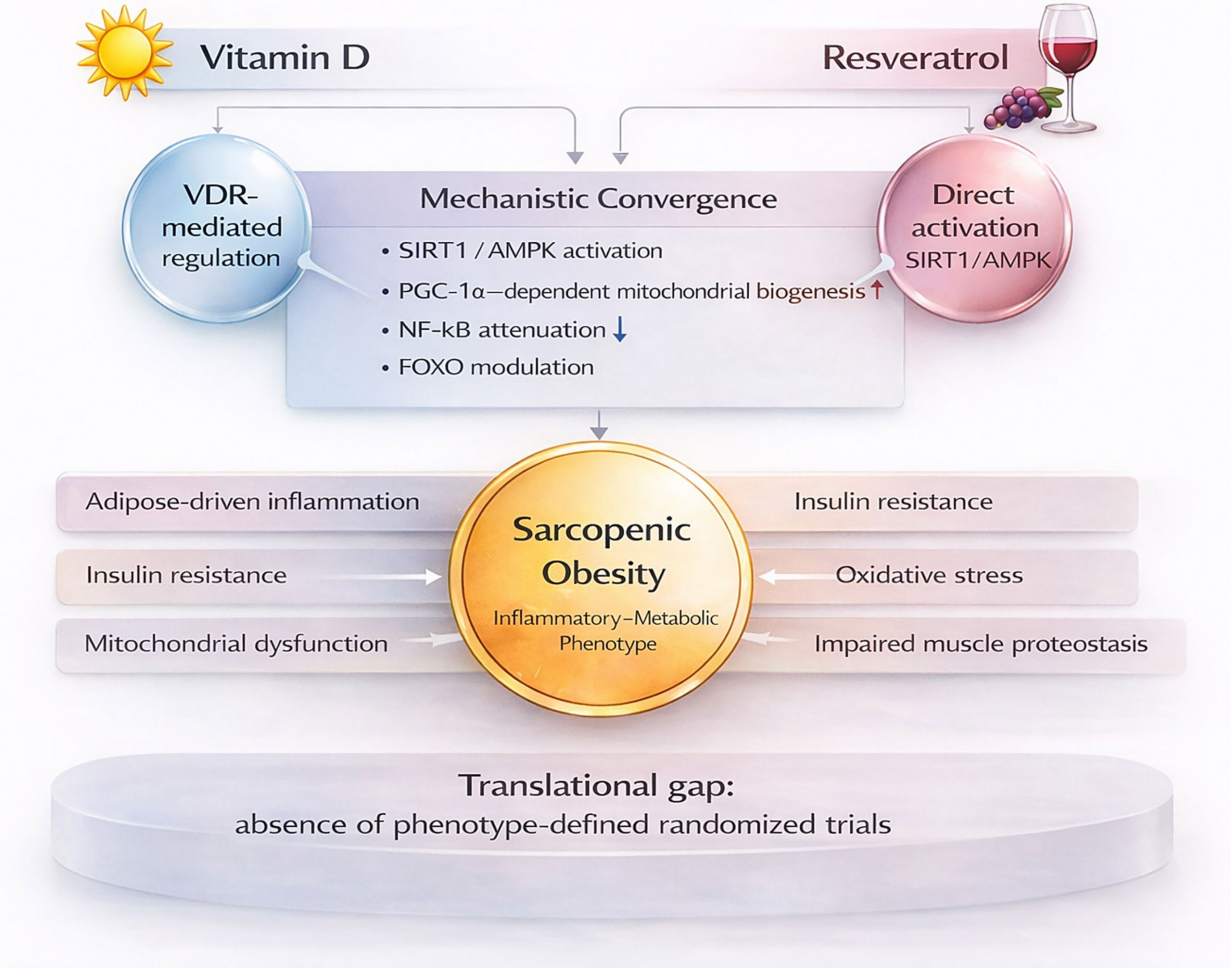
Molecular convergence of vitamin D and resveratrol in sarcopenic obesity and the translational gap in phenotype-defined trials. Vitamin D and resveratrol enter through distinct upstream mechanisms, VDR-mediated genomic regulation and SIRT1/AMPK activation, respectively, but converge on shared downstream signaling pathways, including PGC-1α-dependent mitochondrial biogenesis, NF-κB attenuation, and FOXO modulation. These pathways intersect with the inflammatory-metabolic disturbances characteristic of sarcopenic obesity. Despite this biological coherence, no phenotype-defined randomized controlled trials have evaluated isolated supplementation strategies. VDR, vitamin D receptor; SIRT1, sirtuin 1; AMPK, AMP-activated protein kinase; PGC-1α, peroxisome proliferator-activated receptor gamma coactivator 1-alpha; NF-κB, nuclear factor kappa B; FOXO, forkhead box O transcription factors.

This systematic review sought to determine whether the mechanistic convergence between vitamin D and resveratrol has translated into phenotype-defined randomized clinical investigation in SO. Despite a clear biological rationale, no RCTs were identified that evaluated isolated supplementation of either compound in populations rigorously defined by the concurrent presence of excess adiposity and objective sarcopenia (16–20). The absence of eligible trials reflects a structural gap in trial design rather than evidence of inefficacy. SO represents an integrated inflammatory-metabolic condition characterized by adipose tissue-driven inflammation, insulin resistance, oxidative stress, mitochondrial dysfunction, and impaired muscle proteostasis (5, 21). Within this complex biological environment, vitamin D and resveratrol appear to modulate partially overlapping pathways involved in cellular energy sensing and inflammatory regulation, although these effects arise through distinct upstream mechanisms. Experimental and early clinical data indicate that both compounds influence SIRT1/AMPK signaling, promote PGC-1α-dependent mitochondrial biogenesis, attenuate NF-κB activation, and modulate FOXO activity, thereby contributing to redox balance and metabolic regulation (22–24). These downstream targets are directly relevant to the pathophysiological features of SO. The principal distinction between the two compounds lies in their primary regulatory entry point rather than in their downstream molecular effects. Vitamin D acts predominantly through ligand-dependent activation of VDR, mediating genomic transcriptional regulation that interfaces with inflammatory and mitochondrial pathways (25). Beyond its classical skeletal actions, vitamin D has also been associated with modulation of systemic metabolic and inflammatory biomarkers in human populations, including improvements in inflammatory signaling and cardiometabolic risk profiles (27–30). Resveratrol, by contrast, exerts its effects through direct activation of SIRT1 and AMPK, initiating metabolic and redox adaptations via NAD^+^-dependent deacetylase activity (26). These mechanistic observations support the biological plausibility of investigating both agents within the SO phenotype. However, the current clinical literature remains methodologically fragmented. Trials conducted in obesity frequently assess body composition or functional outcomes without requiring baseline confirmation of sarcopenia, whereas trials focused on sarcopenia rarely mandate adiposity criteria or stratify participants by metabolic burden (26). Even when both adiposity and muscle-related measures are reported, enrollment is seldom based on predefined combined diagnostic thresholds. As a result, studies addressing obesity and sarcopenia are often conducted in parallel rather than within an explicitly defined combined phenotype, which may obscure phenotype-specific responsiveness and contribute to inconsistent findings across intervention studies (22). The five phenotype-adjacent randomized trials identified in this review illustrate this pattern (16–20). Although elements of adiposity and muscle function were measured, none operationalized SO as a formal inclusion criterion while simultaneously isolating vitamin D or resveratrol supplementation. Although, investigative activity exists explicit phenotype alignment is largely absent, and the translational pathway from molecular rationale to clinical validation remains incomplete. From a clinical standpoint, the current data do not support routine use of vitamin D or resveratrol specifically for SO beyond established indications such as correction of vitamin D deficiency. Assessment of intervention efficacy should be anchored to phenotype-relevant endpoints, including improvements in muscle mass and strength, attenuation of inflammatory and oxidative stress, and restoration of cellular metabolic function. Standardized pre- and post-intervention measures are therefore critical to ensure comparability and interpretability across studies. Nevertheless, the mechanistic overlap between these compounds and the inflammatory-mitochondrial disturbances characteristic of SO suggests that targeted evaluation remains warranted (Figure 2). Responsiveness to micronutrient interventions in this phenotype may depend on baseline inflammatory status, metabolic dysregulation, mitochondrial function, and micronutrient sufficiency rather than chronological age alone. This review has several strengths. The use of a phenotype-sensitive dual-track search strategy allowed capture of both explicitly labeled and implicitly defined SO constructs. The restrictive phenotype-based eligibility criteria were intentionally designed to minimize misclassification bias and avoid overinterpretation of heterogeneous populations. Strict eligibility criteria requiring simultaneous confirmation of adiposity and sarcopenia at baseline ensured methodological consistency and avoided overestimation of available evidence. However, limitations should be acknowledged. The requirement for explicit baseline confirmation may have excluded studies with incomplete reporting, and heterogeneity in operational definitions of sarcopenia and adiposity across trials complicates comparisons. Importantly, the absence of eligible trials precluded quantitative synthesis. This absence should be interpreted as a true gap in phenotype-defined randomized evidence rather than a limitation of the review approach, reflecting instead the structure and reporting of the current evidence base. In summary, there is currently no randomized interventional evidence evaluating isolated vitamin D or resveratrol supplementation in rigorously defined SO. Although both compounds influence regulatory pathways associated with mitochondrial function, inflammatory signaling, and metabolic regulation (23), their investigation within phenotype-defined clinical frameworks remains limited. Future randomized trials should incorporate explicit baseline confirmation of both adiposity and muscle impairment, alongside mechanistic endpoints aligned with inflammatory and mitochondrial pathways, to determine whether modulation of micronutrient signaling can meaningfully influence outcomes in SO.

## 5 Conclusion and future directions

No RCTs have evaluated isolated vitamin D or resveratrol supplementation in rigorously defined SO. This absence of phenotype-specific interventional evidence represents a critical gap in translational research and underscores the need for a coordinated preclinical and clinical agenda. Future preclinical studies should employ integrated models that simultaneously reproduce excess adiposity and impaired muscle function to more accurately mirror the inflammatory-metabolic complexity of SO (27). Within such models, targeted modulation of VDR-mediated transcription and SIRT1/AMPK signaling should be systematically evaluated to determine their impact on mitochondrial biogenesis, inflammatory signaling, redox homeostasis, and muscle proteostasis. Particular attention should be given to the to key downstream pathways including PGC-1α activation, NF-κB attenuation, and FOXO regulation, which represent biologically coherent therapeutic targets in this condition. Clinical investigation must follow a similarly structured approach. Randomized trials should require explicit baseline confirmation of both adiposity and sarcopenia and evaluate vitamin D and resveratrol as isolated interventions to establish their independent efficacy. Although evidence specifically addressing SO remains absent, recent investigations suggest that vitamin D supplementation may influence inflammatory, glicemic, lipid, and blood pressure-related biomarkers in humans, supporting the bilogical plasusibility of further phenotype-defined investigation in SO settings (28–31). Future research should adopt standardized eligibility criteria requiring simultaneous confirmation of excess adiposity and sarcopenia, ensuring methodological consistency across trials and improving the accuracy of translation from preclinical models to clinical investigation. Bridging the gap between mechanistic insights and phenotype-defined clinical trial design, therefore, represents a key translational priority in SO research. Advancing the field will require adequately powered, phenotype-defined trials incorporating functional performance outcomes, body composition measures, and mechanistic biomarkers. Beyond vitamin D and resveratrol, this framework should be extended to additional bioactive compounds targeting shared inflammatory–mitochondrial pathways in sarcopenic obesity, including mitochondrial biogenesis, NF-κB signaling, and FOXO-mediated muscle proteostasis, with the aim of identifying further therapeutically relevant candidates with mechanistic coherence. Only through such translationally aligned research can the biological rationale linking vitamin D and resveratrol to inflammatory-mitochondrial regulation be effectively translated into evidence-based therapeutic strategies for SO.

## Data Availability

The original contributions presented in the study are included in the article/Supplementary material, further inquiries can be directed to the corresponding author.
