# Supplementary material for: Vitamin D and resveratrol in sarcopenic obesity: a systematic review highlighting the gap in phenotype-defined randomized controlled trials
Source: Front Nutr. 2026 May 14;13:1818450. doi: 10.3389/fnut.2026.1818450 (PMC13216041; doi:10.3389/fnut.2026.1818450)
Supplement: Supplementary file 1 [file Table_1.docx]

**Supplementary Table S1. Final Electronic Search Strategies**

| **Database** | **Final Search Strategy** | **Filters Applied** | **Records Retrieved** |
| --- | --- | --- | --- |
| **PubMed (MEDLINE)** | ("sarcopenic obesity"[tiab] OR (sarcopenia[tiab] AND obesity[tiab])) AND ("vitamin D"[tiab] OR cholecalciferol[tiab] OR ergocalciferol[tiab] OR calcifediol[tiab] OR calcitriol[tiab] OR alfacalcidol[tiab] OR paricalcitol[tiab] OR resveratrol[tiab] OR "trans-resveratrol"[tiab]) AND (randomized controlled trial[pt] OR randomized[tiab] OR placebo[tiab]) NOT (review[pt] OR protocol[ti]) | Humans; RCT filter | 5 |
| **Scopus** | TITLE-ABS-KEY(("sarcopenic obesity" OR (sarcopenia AND obesity)) AND ("vitamin D" OR cholecalciferol OR ergocalciferol OR calcifediol OR calcitriol OR alfacalcidol OR paricalcitol OR resveratrol OR "trans-resveratrol") AND (random* OR placebo)) AND DOCTYPE(ar) | Article; randomized terms | 0 |
| **Web of Science (Core Collection)** | TS=(("sarcopenic obesity" OR (sarcopenia AND obesity)) AND ("vitamin D" OR cholecalciferol OR ergocalciferol OR calcifediol OR calcitriol OR alfacalcidol OR paricalcitol OR resveratrol OR "trans-resveratrol") AND (randomized OR placebo)) | Article | 0 |

Searches were conducted from database inception to 31 January 2026. No date restrictions were applied. Syntax was adapted to each database platform. Title/abstract or topic field restrictions were used to ensure explicit capture of phenotype and intervention terms. Review articles and study protocols were excluded. Duplicate removal was performed prior to screening.
